# A tumor cord model for Doxorubicin delivery and dose optimization in solid tumors

**DOI:** 10.1186/1742-4682-6-16

**Published:** 2009-08-09

**Authors:** Steffen Eikenberry

**Affiliations:** 1 Department of Mathematics and Statistics, Arizona State University, Tempe, AZ 85287, USA

## Abstract

**Background:**

Doxorubicin is a common anticancer agent used in the treatment of a number of neoplasms, with the lifetime dose limited due to the potential for cardiotoxocity. This has motivated efforts to develop optimal dosage regimes that maximize anti-tumor activity while minimizing cardiac toxicity, which is correlated with peak plasma concentration. Doxorubicin is characterized by poor penetration from tumoral vessels into the tumor mass, due to the highly irregular tumor vasculature. I model the delivery of a soluble drug from the vasculature to a solid tumor using a tumor cord model and examine the penetration  of doxorubicin under different dosage regimes and tumor microenvironments.

**Methods:**

A coupled ODE-PDE model is employed where drug is transported from the vasculature into a tumor cord domain according to the principle of solute transport. Within the tumor cord, extracellular drug diffuses and saturable pharmacokinetics govern uptake and efflux by cancer cells. Cancer cell death is also determined as a function of peak intracellular drug concentration.

**Results:**

The model predicts that transport to the tumor cord from the vasculature is dominated by diffusive transport of free drug during the initial plasma drug distribution phase. I characterize the effect of all parameters describing the tumor microenvironment on drug delivery, and large intercapillary distance is predicted to be a major barrier to drug delivery. Comparing continuous drug infusion with bolus injection shows that the optimum infusion time depends upon the drug dose, with bolus injection best for low-dose therapy but short infusions better for high doses. Simulations of multiple treatments suggest that additional treatments have similar efficacy in terms of cell mortality, but drug penetration is limited. Moreover, fractionating a single large dose into several smaller doses slightly improves anti-tumor efficacy.

**Conclusion:**

Drug infusion time has a significant effect on the spatial profile of cell mortality within tumor cord systems. Therefore, extending infusion times (up to 2 hours) and fractionating large doses are two strategies that may preserve or increase anti-tumor activity and reduce cardiotoxicity by decreasing peak plasma concentration. However, even under optimal conditions, doxorubicin may have limited delivery into advanced solid tumors.

## Background

Doxorubicin (adriamycin) is a first line anti-neoplastic agent used against a number of solid tumors, leukemias, and lymphomas [1]. There are many proposed mechanisms by which doxorubicin (DOX) may induce cellular death, including DNA synthesis inhibition, DNA alkylation, and free radical generation. It is known to bind to nuclear DNA and inhibit topoisomerase II, and this may be the principle mechanism [2]. Cancer cell mortality has been correlated with both dose and exposure time, and El-Kareh and Secomb have argued that it is most strongly correlated with peak intracellular exposure [3,4]; rapid equilibrium between the intracellular (cytoplasmic) and nuclear drug has been suggested as a possible mechanism for this observation [4].

The usefulness of doxorubicin is limited by the potential for severe myocardial damage and poor distribution in solid tumors [1,5]. Cardiotoxicity limits the lifetime dose of doxorubicin to less than 550 mg/m^2 ^[1,6] and has motivated efforts to determine optimal dosage regimes. Determining optimal dosage is complicated by the disparity in time-scales involved: doxorubicin clearance from the plasma, extravasation into the extracellular space, and cellular uptake all act over different time-scales. A mathematical model by El-Kareh and Secomb [3] took this into account and explicitly modeled plasma, extracellular, and intracellular drug concentrations. They compared the efficacy of bolus injection, continuous infusion, and liposomal delivery to tumors. They took peak intracellular concentration as the predictor of toxicity and found continuous infusion in the range of 1 to 3 hours to be optimal. However, this work considered a well-perfused tumor with homogenous delivery to all tumor cells. Optimization of doxorubicin treatment is further complicated by its poor distribution in solid tumors and limited extravasation from tumoral vessels into the tumor extracellular space [5,7]. Thus, the spatial profile of doxorubicin penetrating into a vascular tumor should also be considered.

Most solid tumors are characterized by an irregular, leaky vasculature and high interstitial pressure. In most tumors capillaries are much further apart than in normal tissue. This geometry severely limits the delivery of nutrients as well as cytotoxic drugs [5]. There has been significant interest in modeling fluid flow and delivery of macromolecules within solid tumors [8-11]. Some modeling work has considered spatially explicit drug delivery to solid tumors [12-14], El-Kareh and Secomb considered the diffusion of cisplatin into the peritoneal cavity [15], and doxorubicin has attracted significant theoretical attention from other authors [16-18].

I propose a model for drug delivery to a solid tumor, considering intracellular and extracellular compartments, using a tumor cord as the base geometry. Tumor cords are one of the fundamental microarchitectures of solid tumors, consisting of a microvessel nourishing nearby tumor cells [13]. This simple architecture has been used by several authors to represent the *in vivo *tumor microenvironment [13,19], and a whole solid tumor can be considered an aggregation of a number of tumor cords. Plasma DOX concentration is determined by a published 3-compartment pharmacokinetics model [20], and the model considers drug transport from the plasma to the extracellular tumor space. The drug flux across the capillary wall takes both diffusive and convective transport into account, according to the principle of solute transport [21]. The drug diffuses within this space and is taken up according to the pharmacokinetics described in [3]. Doxorubicin binds extensively to plasma proteins [22], and therefore both the bound and unbound populations of plasma and extracellular drug are considered separately.

Using this model, I predict drug distribution within the tumor cord and peak intracellular concentrations over the course of treatment by bolus and continuous infusion. Cancer cell death as a function of peak intracellular concentration over the course of treatment by continuous infusion is explicitly determined according to the *in vitro *results reported in [23]. The roles of all parameters describing DOX pharmacokinetics and the tumor microenvironment are characterized through sensitivity analysis.

The model is applied to predicting the efficacy of different infusion times and fractionation regimes, as well as low versus high dose chemotherapy. Continuous infusion is compared to bolus injection, and I find that the continuous infusions on the order of 1 hour or less can slightly increase maximum intracellular doxorubicin concentration near the capillary wall and have similar overall cancer cell mortality. Optimal infusion times depend upon the dose, with rapid bolus more efficacious for small doses (25–50 mg/mm^2^) and short infusions better for higher doses (75–100 mg/mm^2^). Fractionating single large bolus injections into several smaller doses can also slightly increase efficacy. Cardiotoxicity is correlated with peak plasma AUC [24], and even relatively brief continuous infusions or divided dosages greatly reduce peak plasma concentration. Therefore, such infusion schedules likely preserve or even enhance anti-tumor activity while reducing cardiotoxicity.

I examine the efficacy of high dose versus low dose chemotherapy, finding that cytotoxicity at the tumor vessel wall levels off with increasing doses, but overall mortality increases nearly linearly. However, when the tumor intercapillary distance, and hence tumor cord radius, is large, even extremely high doses fail to cause significant mortality beyond 100 *μ*m from the vessel wall. Multiple treatments are also simulated, and drug penetration is limited even after several treatments. Therefore, the model predicts that DOX delivery to advanced tumors may be limited. 

Techniques to evaluate the penetration of drugs *in vivo *are technically challenging [5], but traditional *in vitro *experiments fail to give a complete understanding of drug activity *in vivo *[5,7]. Adapting experimental results concerning the effects of intracellular drug concentration (as in [23]) and the tumor microenvironment on cell death to a theoretical framework that models an *in vivo *tumor is a promising avenue of investigation into the optimization of drug dosage regimes.

## Methods

### Tumor cord model

**Figure 1:**
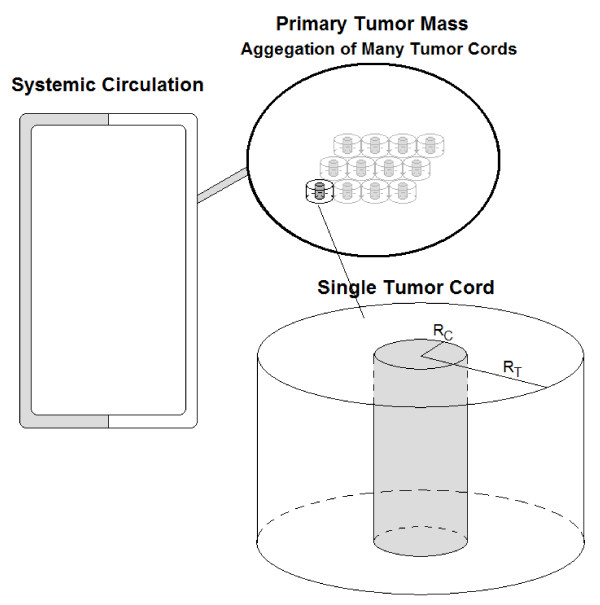
**The modeled tumor system**. The systemic circulation is connected to the primary tumor mass. The primary mass is composed of a number of individual tumor cords. Doxorubicin delivery is considered in one of these tumor cords.

I assume a tumor cord geometry with both axial and radial symmetry. Therefore, the three-dimensional problem can be considered with only one variable for the radius – *r*. The capillary wall extends to *R*_*C*_, and the tumor cord extends to a radius of *R*_*T*_. I also assume that cancer cell density is uniform throughout the tumor cord and that all cells are viable. I do not consider the effects of hypoxia or necrotic areas distant from the capillary. This is a reasonable approximation, as in a study of doxorubicin concentration in solid tumors by Primeau *et al. *[7], drug concentration decreased exponentially with distance from blood vessels. Drug concentration was reduced by half at 40–50 *μ*m from vessels, but the distance to hypoxic regions was reported as 90–140 *μ*m. A negligible amount of drug reached the hypoxic region, while many viable cells were unaffected. Therefore, in this study, it is not necessary to consider the effects of hypoxia, and I only consider the viable part of the tumor cord. A schematic of the circulation coupled to the tumor cord system as modeled is shown in Figure 1.

The model considers plasma, free extracellular, albumin-bound extracellular, and intracellular drug concentration as four separate variables. Plasma drug concentration is determined according to a 3-compartment pharmacokinetics model, based on the previously published model of Robert *et al. *[20]. Transport of drug from plasma into the tumor extracellular space occurs by passive diffusion and convective transport across the capillary wall according to the Staverman-Kedem-Katchalsky equation [21]. For some general solute, *S*, the transcapillary flux is given as:

(1)

(2)

where *S*_*V *_is the solute concentration on the vascular side of the capillary and *S*_*E *_is the concentration on the extracellular side. The first term gives transport by diffusion, and the second is transport by convection. *P *is the diffusional permeability coefficient, *A *is the capillary surface area for exchange, *σ*_*F *_is the solvent-drag reflection coefficient, Δ*S*_*lm *_is the log-mean concentration difference, and *J*_*F *_is the fluid flow as given by Starling's hypothesis:

(3)

Here, *L*_*p *_is the hydraulic conductivity, *P*_*V*_-*P*_*E *_is the hydrostatic pressure difference, Π_*V*-_Π_*E *_is the osmotic pressure difference, and *σ *is the osmotic reflection coefficient. The applications of these equations to this particular model are given below.

Once extravasation into the extracellular space has occurred, the drug diffuses by simple diffusion. Bound and unbound drug are transported across the vessel wall independently. Within the extracellular space, the two populations diffuse at different rates, and drug rapidly switches between the bound and unbound states. Changes in extra and intracellular drug concentrations are governed by the pharmacokinetics model described in [3], which assumes Michaelis-Menten kinetics for doxorubicin uptake. Transport of doxorubicin across the cell membrane is a saturable process [25], yet actual transport across the membrane occurs by simple Fickian diffusion [26]. This apparent paradox has been explained by the ability of doxorubicin molecules to self-associate into dimers that are impermeable to the lipid membrane, causing transport to mimic a carrier-mediated process [23,26]. A later model by El-Kareh and Secomb [4] additionally considered non-saturable diffusive transport, but this process is of less importance, and I disregard it in this model.

I assume that over the course of a single treatment no drug-induced cell death occurs, implying that cancer cell density is constant in time. Cancer cell density is also assumed to be (initially) homogenous throughout the tumor cord. However, when considering multiple treatments, the spatial profile of cancer cells is updated between treatments, as is the fraction extracellular space. The peak intracellular drug concentration over the course of a treatment is tracked. At the end of this time, likely cell death is determined according to the peak intracellular drug concentration vs. surviving fraction for doxorubicin given in [23]. The model variables are:

1. *C*(*r*) = Cancer cell density (cells/mm^3^)

2. *S*(*t*) = Plasma drug concentration (*μ*g/mm^3^)

3. *F*(*r, t*) = Free extracellular drug concentration (*μ*g/mm^3^)

4. *B*(*r, t*) = Bound extracellular drug concentration (*μ*g/mm^3^)

5. *I*(*r, t*) = Intracellular drug concentration (ng/10^5 ^cells)

Some care must be taken concerning the units for *F *and *B*, which represent the concentration in *μ*g per mm^3 ^of space. This space includes all tissue, not just the space that is explicitly extracellular. The fraction of space that is extracellular is represented by *ϕ*. Moreover, *B *refers strictly to the concentration of bound doxorubicin in *μ*g/mm^3^, i.e. the albumin component of the albumin:DOX complex is not considered in the units of concentration, so 1 *μ*g/mm^3 ^of free DOX corresponds directly to 1 *μ*g/mm^3^of bound DOX. However, the properties of the albumin:DOX complex (MW, etc.) must still be taken into account in parametrization.

A number of 2- and 3-compartment pharmacokinetics models for plasma doxorubicin concentration have been proposed [20,22,24]. The plasma kinetics are largely describable with a 2-compartment model. The initial distribution phase is characterized by a very short half-life (5–15 min), while the half-life of elimination is on the order of a day (18–35 hrs). However, some authors have achieved a better fit to the data using a 3-compartment model. Robert *et al. *[20] determined pharmacokinetic parameter using a 3-compartment model for 12 patients with unresectable breast cancer; Eksborg *et al. *[24] also reported similar pharmacokinetic parameters for a 3-compartment model for 21 individual patients. Therefore, I use the following 3-compartment model for plasma concentration that can be described using differential equations as

(4)

(5)

(6)

(7)

That is, total plasma concentration, *S*(*t*), is the sum of 3 compartments *C*_1_(*t*), *C*_2_(*t*), and *C*_3_(*t*). Here, *D *is the total dose (*μ*m) injected and *T *is the infusion time (3 minutes for a rapid bolus). The Heaviside term *H*(*T*-*t*) indicates that infusion only occurs between *t *= 0 and *t *= *T*. This formulation is useful for simulating multiple infusions of drug when complete clearance between infusions has not occurred. The plasma concentration for a single infusion may also be given explicitly as

(8)

when *t *<*T*, and

(9)

when *t *≥ *T*.

The PDE component of the model governs dynamics within the spatial environment of the tumor cord as follows:

(10)

(11)

(12)



Boundary conditions are used to account for an influx of doxorubicin at the capillary wall:



(13)

(14)





No-flux boundary conditions are used for all variables at the outer radius of the tumor cord. The drug fluxes per unit area across the capillary wall are *J*_Free _and *J*_Bound_. In each, the first term gives the rate of passive diffusion due to concentration differences in the blood and extracellular drug compartments. The second term represents drug transported by convective forces. Blood concentration and serum concentration are not identical; the blood concentration is *θS*, where *θ *is the fraction of blood that is plasma (0.6). Likewise, *F *is the concentration of free doxorubicin per mm^3 ^of tissue space, while *F*/*ϕ *is the concentration in the extracellular space. The fraction of tissue adjacent to the capillary wall that is extracellular space is *ϕ*, implying that the effective concentration of drug on the tissue side of the capillary wall is *ϕ *× *F*/*ϕ *= *F*. Thus, the flux of free drug is a function of *θ (1-δ) S *and *F*, where *δ *is the fraction of plasma drug bound to albumin. The flux of bound drug is similarly a function of *θδS *and *B*. There are two versions for all transport parameters, one for free DOX (typically subscripted by *F*) and one for bound DOX (subscripted by *B*). Note that the exception is the solvent-drag reflection coefficient, which is generally given as *σ*_*F*_, so *F *and *B *are superscripted for this parameter.

The cellular uptake and efflux functions are *μ *and *υ*, respectively. These are similar to those used in [3], and *V*_max _gives the maximum rate of transport in terms of ng/(10^5 ^cells hr). *K*_*E *_and *K*_*I *_are the Michaelis constants for half-maximal transport. In the study by Kerr *et al. *[23], from which these functions were determined, cells were cultured in a medium that included foetal calf serum. Therefore, significant albumin was likely present, implying that *K*_*E *_refers to the sum of both bound and unbound drug. However, only unbound doxorubicin is likely to cross the cell membrane. Thus, *μ *depends on both *F *and *B*, but only free drug is actually transported, and *μ *and *υ *only appear in the equation for *F*.

Transport across cell membranes at a given spatial point depends upon drug concentration per mm^3 ^of extracellular space and not general tissue space – the unit for *F *and *B*. This causes the dependence upon *ϕ*, the fraction of space that is extracellular, in the uptake function *μ*. The simple scaling parameter *ρ *is also included to keep units consistent.

Finally, the initial condition for all model variables is 0, except cancer cells, which are initially set to density *d*_*C *_at all points:



### Tumor cell survival

**Figure 2:**
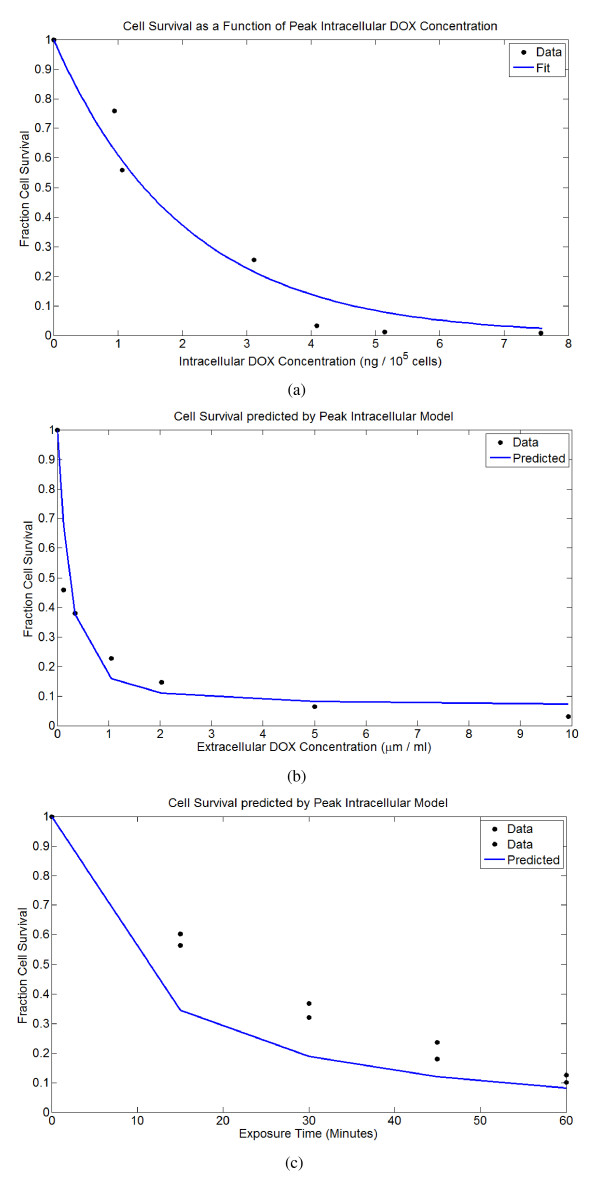
**Cell survival predicted as an exponential function of peak intracellular DOX concentration, using data from Kerr et al. **[23]. Using this fit and the drug uptake model gives good agreement to a second data-set published in the same paper, but a rather poor agreement with a third. (A) Cell survival as a function of intracellular drug concentration. (B) Predicted cell survival versus the actual cell survival for cells exposed to different concentrations of DOX for 1 hour. (C) Predicted cell survival versus the actual cell survival for cells exposed to 5 *μ*m/ml of DOX for 15, 30, 45, and 60 minutes.

It has previously been reported that survival in cancer cells exposed to DOX is an exponential function of the extracellular AUC [22]. However, El-Kareh and Secomb have argued that peak intracellular concentration is a better predictor of cell survival [3,4]. I estimate cancer cell mortality using the *in vitro *data of Kerr *et al. *[23], who found the relationship between intracellular DOX concentration and log cell survival to be linear in non-small cell lung cancer cells. The surviving cell fraction, *S*_*F*_, is determined as an exponential function of peak intracellular DOX concentration:



where *ω *= 0.4938 gives the best fit to the data. Using the pharmacokinetic model for DOX uptake together with this fit gives good agreement for cell survival with a separate data-set published in the same paper, where cells were exposed to different concentrations of DOX for 1 hour. However, this model overestimates mortality for a second data-set where cells were exposed to 5 *μ*m/ml of DOX for shorter periods of time, suggesting that in reality both exposure time and peak concentration are important in determining cytotoxicity. The fit and comparisons are shown in Figure 2.

Because cell survival was assessed using a clonogenic assay, cytotoxicity for an *in vivo *tumor may be overestimated, as a much smaller fraction of cells in an advanced tumor will be proliferating than in such an assay.

### Parametrization

Values for all model parameters can be estimated from empirical biological data and from previous models. I use transport parameters for albumin for the bound doxorubicin and directly determine these parameters for free doxorubicin. The plasma fraction of blood, *θ*, is assumed to be 0.6, and a body surface area of 1.73 m^2 ^is assumed.

#### Tumor cord geometry parameters

##### Vessel and cord radii

Tumors can vary greatly in the level of perfusion and in the regularity of their vasculature. Furthermore, there is great heterogeneity within single tumors [27-29]. Tumoral vasculature is characterized by irregular branching patterns with capillaries arranged in irregular meshworks that were studied in [28]. The mean capillary diameter was measured as 10.3 ± 1.4 *μ*m, and the mean capillary length was 66.8 ± 34.2 *μ*m. Mean vessel diameter for melanoma xenografts varied between 9.5 and 14.6 *μ*m in [29]. However, larger values have been reported, and vessel diameter was 20.0 ± 6.2 *μ*m for neoplastic tissue in [30]. Furthermore, Hilmas *et al. *[31] found that vessel diameter increased dramatically with tumor size, increasing from about 10 *μ*m to over 30 *μ*m.

In [13], for various tumors, the blood vessel radius for tumor cords was reported as 10–40 *μ*m and the viable tumor cord radius was 60–130 *μ*m from the vessel wall. The mean tumor cord radius for squamous cell carcinomas was measured as 104 *μ*m in [32]. Primeau *et al. *[7] measured the mean distance from vessels to hypoxic regions as 90–140 *μ*m.

##### Capillary surface area

Total capillary surface area varies greatly between tumor types and individual tumors. Surface areas were measured as 1.2–2.6 × 10^4^[31], 1.5–5.7 × 10^4^, and 0.5–2.0 × 10^4 ^mm^2^/g wet wt. [21] for mouse mammary carcinomas, mouse mammary adenocarcinomas, and rat hepatomas. Larger tumors typically have less vascular surface area [21], although vascular volume may stay relatively constant [31].

##### Fraction extracellular space

The fraction of extracellular space, *ϕ*, in tumors is much greater than in normal tissue and may range from 0.2 to 0.6 [33]. Assuming that average tumor cell diameter ranges between 10 and 20 *μ*m, tumor cell density may range from as little as 0.955 × 10^5 ^cells/mm^3 ^to as much as 1.53 × 10^6 ^cells/mm^3 ^(assuming *ϕ *between 0.2 and 0.6).

#### Transport parameters

##### Hydrostatic fluid pressures (*P*_*V*_, *P*_*E*_)

Tumor capillary fluid pressures (parameter *P*_*V*_) range roughly from 10 to 30 mmHg, and interstitial fluid pressure (IFP, parameter *P*_*E*_) within the tumor is often close to or even greater than fluid pressure within the capillary [21,29]. For example, Boucher and Jain [34] found rat mammary adenocarcinoma microvessel pressures to range from 7–31 mmHg (17.3 ± 6.1 mmHg) and tumor IFP ranged 4.4–31.5 mmHg (18.4 ± 9.3 mmHg). The greatest pressure drop was 7 mmHg, and the fluid pressure in the vessel was usually greater than in the interstitium, although in some cases the IFP was greater. The IFP in the outer region is typically much lower than the central region [34,35], and larger tumors have greater IFP everywhere [21].

##### Osmotic pressures (Π_*V*_, Π_*E*_)

In most species, the plasma osmotic pressure is about 20 mmHg [36]. Due to the leaky nature of tumor vessels, many macromolecules are present in the interstitium, and osmotic pressure in tumoral tissue is near that of the plasma. In [36], Π_*V *_= 20.0 ± 1.6 mmHg, and Π_*E *_= 16.7 ± 3.0, 19.9 ± 1.9, 21.8 ± 2.8, and 24.2 ± 4.7 mmHg for colon adenocarcinoma, squamous cell carcinoma, small cell lung carcinoma, and rhabdomyosarcoma mouse xenografts, respectively. Thus, while often ΔΠ ≈ 0, a reasonable range is ΔΠ = -9.0 – 8.0 mmHg.

##### Osmotic reflection coefficient (*σ*)

It is assumed that macromolecules such as albumin are the dominant contributors to the osmotic pressure gradient between the vessel and tumor tissue. The osmotic reflection coefficient for albumin, *σ*, is between .8 and .9 in most tissues, and approaches 1 in skeletal muscle and the brain [21].

##### Solvent-drag reflection coefficients (, )

The solvent-drag reflection coefficient, , for albumin was measured at .82 ± .08 in the perfused cat hindlimb [37]. Osmotic reflection and solvent-drag reflection coefficients were similar in [38], and *σ*_*F *_≃ *σ *in dilute solutions [21]. In [39], *σ *= .35 ± .16 for raffinose in dog lung endothelium, and since the molecular weight of raffinose (504) is similar to that of doxorubicin (544), I let  = .35.

##### Hydraulic conductivity (*L*_*p*_)

Sevick and Jain [40] measured the capillary filtration coefficient (CFC), i.e. *L*_*p*_*A *where *A *= vascular surface area, for mouse mammary adenocarcinomas, finding CFC ≈ 2.6 ± .5 ml/. Using vascular surface areas for mouse mammary tumors (*A *= 1.2 – 5.7 × 10^4 ^mm^2^/g wet wt) allows *L*_*p *_to be estimated as .022–.16 mm^3^/hr/mmHg.

##### Diffusional permeability (*P*_*F*_, *P*_*B*_)

Estimating the vascular permeability coefficient, *P*, is complicated by the fact that most estimates are of the "effective permeability coefficient," *P*_Eff_, which subsumes both diffusive and convective transport into a single parameter. In tumoral tissue, this may be close to the actual permeability coefficient if both osmotic and hydraulic pressures are similar within plasma and the interstitium, which is typically the case [34]. Wu *et al. *[41] measured *P*_Eff _for albumin to be about three-fold higher in tumoral compared to normal tissue, and Gerlowski and Jain [30] found *P*_Eff _to be 8 times higher for 150 KDa dextran in tumor tissue. Using published values for *P*_Eff _for molecules with MWs similar to DOX and these ratios, I estimate that for free DOX, *P*_Eff _= 2.916 – 13.306 mm/hr [41,42]. Ribba *et al. *[17] used *P *= 10.8 mm/hr for DOX in a mathematical model. Wu *et al. *[41] measured *P*_*Eff *_= .0281 ± .00432 mm/hr for albumin (corresponding to albumin-bound DOX) in tumor tissue, although the authors considered this to be an underestimate. Such measurements for *P*_Eff _give a high but not unrealistic estimate for the actual *P*, as convective flux is considered to be minimal in most tumors [34].

I note that capillary fenestration dramatically increases permeability for small molecules, but does not appear to significantly affect macromolecules [43]. Fenestration may increase hydraulic conductivity 20-fold [43] and, for molecules similar in size to free DOX, the effective permeability coefficient may be 2 orders of magnitude higher [21].

##### Diffusion coefficients (*D*_*F*_, *D*_*B*_)

Based on the relationship given in [44] (*D *= .0001778 × (MW)^-.75^), the diffusion coefficient for free extracellular doxorubicin, *D*_*F*_, is calculated to be 0.568. However, it may be significantly higher, as Nugent and Jain [33] found that the diffusion coefficient for small molecules in tumor tissue was nearly that predicted by the Einstein-Stokes relation for free diffusion in water (*D*_0_). McLennon *et al. *[45] estimated a molecular radius of 3 Å for daunomycin, which implies *D*_0 _= 4.03 mm^2^/hr. Assuming *D*/*D*_0 _is at most 0.89 [33], *D*_*F *_may be as great as 3.587 mm^2^/hr.

Diffusion of macromolecules is significantly higher in tumoral than in normal tissue [21,33]. The effective diffusion coefficient for albumin in VX2 carcinoma was measured as .03276 mm^2^/hr [33], about twice that predicted by the relation in [44] (.01537 mm^2^/hr). Using the FRAP technique, Chary and Jain [46] estimated a diffusion coefficient an order of magnitude higher at .2268 mm^2^/hr, but stated that this technique likely measures diffusion in the fluid phase of the interstitium, rather than the effective diffusion coefficient. But, since tumors have a very large fraction extracellular space, the effective diffusion coefficient may still be close to this value.

#### Pharmacokinetics parameters

Most doxorubicin is bound to plasma proteins. Greene *et al. *[22] found 74–82% to be bound; the percentage bound was independent of both doxorubicin and albumin concentration. Wiig *et al. *[47] found albumin concentration to be high in rat mammary tumor interstitial fluid at 79.9% of the plasma concentration. Therefore, it is likely that doxorubicin-albumin binding in the tumor extracellular space is similar to that in plasma. I assume that the on/off binding kinetics of free and bound DOX in the are fast relative to the other processes in the model and take *k*_*d *_/*k*_*a *_= (fraction free), with *k*_*d *_and *k*_*a *_large.

The pharmacokinetic parameters *V*_*max*_, *K*_*E*_, and *K*_*I*_, were determined by El-Kareh and Secomb in [3] using data given by Kerr *et al. *[23]. The cell mortality constant *ω *has been determined using data from the same paper as shown in Figure 2. Table 1 gives all parameters, values, and references used.

**Table 1:** All parameters and values.

**Parameter**	**Meaning**	**Value**	**Reference**
*A*	Compartment 1 parameter	15.7–-130.3 × 10^-9 ^mm^-3 ^(74.6 × 10^-9^)	[20]
*B*	Compartment 2 parameter	.415–-6.58 × 10^-9 ^mm^-3 ^(2.49 × 10^-9^)	[20]
*C*	Compartment 3 parameter	.277–-.977 × 10^-9 ^mm^-3^(.552 × 10^-9^)	[20]
*α*	Compartment 1 clearance rate	5.09–12.76/hr (9.68)	[20]
*β*	Compartment 2 clearance rate	.520–2.179/hr (1.02)	[20]
*γ*	Compartment 3 clearance rate	.0196–.0804/hr (.0423)	[20]
			
*V*_*max*_	Rate for transmembrane transport	16.8 ng/(10^5 ^cells hr)	[3]
*K*_*E*_	Michaelis constant	2.19 × 10^-4 ^*μ*g/mm^3^	[3]
*K*_*I*_	Michaelis constant	1.37 ng/10^5 ^cells	[3]
			
*ρ*	Scaling factor	10^-8 ^*μ*g (10^5 ^cells)/(ng cell)	
*ϕ*	Tumor fraction extracellular space	0.2–0.6 (0.4)	[33]
*d*_*C*_	Density of tumor cells	0.955–-15.3 × 10^5 ^cells/mm^3 ^(10^6^)	see text
*D*_*F*_	Free DOX diff. coeff.	0.568–3.587 mm^2^/hr (.568)	[33,44,45]
*D*_*B*_	Bound DOX diff. coeff.	.03276–.2268 mm^2^/hr (.032)	[33,46]
*P*_*F*_	Diffusive permeability for free DOX	2.916–13.306 mm/hr (10.0)	[41,42]
*P*_*B*_	Diffusive permeability for bound DOX	.02378–.03242 mm/hr (.032)	[41]
			
*P*_*V*_	Tumor capillary fluid pressure	4.4–31.5 mmHg (20.0)	[34]
*P*_*E*_	Tumor IFP	4.4–31.5 mmHg (15.0)	[34]
*L*_*p*_	Hydraulic conductivity	.022–.16 mm^3^/hr/mmHg (0.1)	[21,31,40]
*σ*	Osmotic reflection coefficient	.8–1.0 (.85)	[21]
	Coupling coefficient for free DOX	.19–.51 (.35)	[21,38,39]
	Coupling coefficient for bound DOX	.74–.9 (.82)	[37,38]
Π_*V*_	Plasma colloid osmotic pressure	20 mmHg	[36]
Π_*E*_	Tumor colloid osmotic pressure	13.7–27.9 mmHg (20)	[36]
			
*A*	Total tumor vasculature surface area	0.5–5.7 × 10^4 ^mm^2^/g wet wt.	[21]
*R*_*C*_	Tumor capillary radius	5–20 *μ*m (10)	[13,30,31]
*R*_*T*_	Viable tumor cord radius	50–150 *μ*m (150)	[7,13,28,32]
			
*δ*	Fraction of plasma DOX bound	.74–.82 (.75)	[22]
*k*_*a*_	Free DOX-albumin binding rate	3000–4000/hr (3000)	see text
*k*_*d*_	DOX-albumin dissociation rate	1000/hr	see text
ω	Cell survival exponential constant	0.4938	[23], see text

The possible parameter range as determined in the text is given, and the default value used in simulations is in parentheses.

### Numerical methods

The coupled ODE-PDE system is solved numerically in the tumor cord geometry using an explicit finite difference method for the PDE portion. The ODE system is either solved explicitly as in Equations 8 and 9, or solved numerically using either first-order differencing in time. When simulating multiple treatments, each treatment is run as a separate simulation. The expected cell mortality at every spatial point is then calculated, and this is used to determine a spatial profile of cell density, which is then given as the initial condition for *C*(*r*) for the simulation of the next treatment.

## Results and discussion

### Basic model dynamics

**Figure 3:**
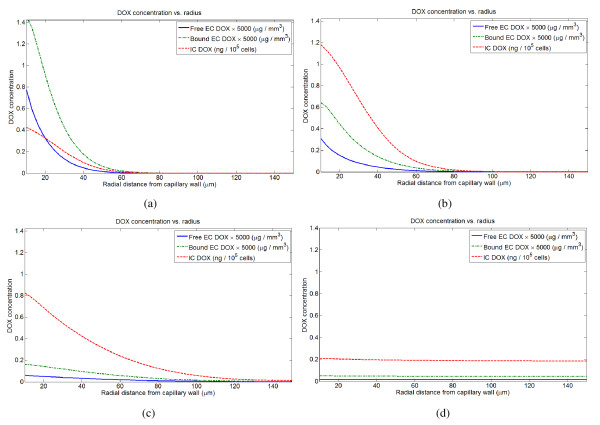
**Intracellular and extracellular doxorubicin distribution in the tumor cord following a 3 minute infusion (rapid bolus) of 105 mg/m^2^**. Profiles are shown at (A) 3 mintues, (B) 10 minutes, (C) 1 hour, (D) 24 hours.

For both rapid bolus and short infusions, the distribution of DOX to tumor cells within the tumor cord occurs in essentially two phases. The first phase roughly corresponds to the plasma distribution (*α*) phase, and in this phase a gradient of both intracellular and extracellular drug is established. In the second phase, corresponding to the plasma elimination (*γ*) phase, intracellular and extracellular concentrations decrease and flatten in space. They also remain nearly static in time, decreasing very slowly compared to the time-scale of the first phase. Eventually, the gradient inverts, and DOX slowly clears from the extracellular space and back into the plasma. Within the tumor cord, most drug is sequestered either in the intracellular compartment or bound to proteins; only a small fraction is free. The first phase is primarily responsible for cell kill within 100 *μ*m of the vessel wall, while the second phase establishes a low, uniform level of mortality throughout the tumor cord. Thus, the first phase is likely dominant in drug delivery to the non-hypoxic portion of the tumor cord, while the second dominates drug penetration deeper within the cord. This pattern of DOX distribution in the tumor cord as a function of time for a rapid bolus is shown in Figure 3.

### Different infusion times and doses

**Figure 4:**
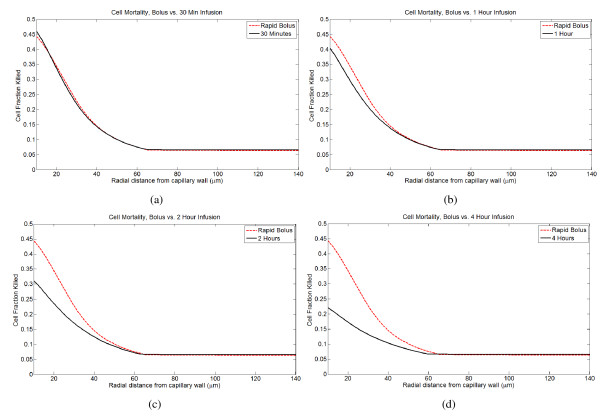
**Spatial profiles of predicted cancer cell mortality under different infusion times**. The results for a rapid bolus (3 minute infusion) are compared to 30, 60, 120, and 240 minute infusions. (A) Rapid bolus vs. 30 minute infusion. (B) Rapid bolus vs. 1 hour infusion. (C) Rapid bolus vs. 2 hour infusion. (D) Rapid bolus vs. 4 hour infusion.

**Figure 5:**
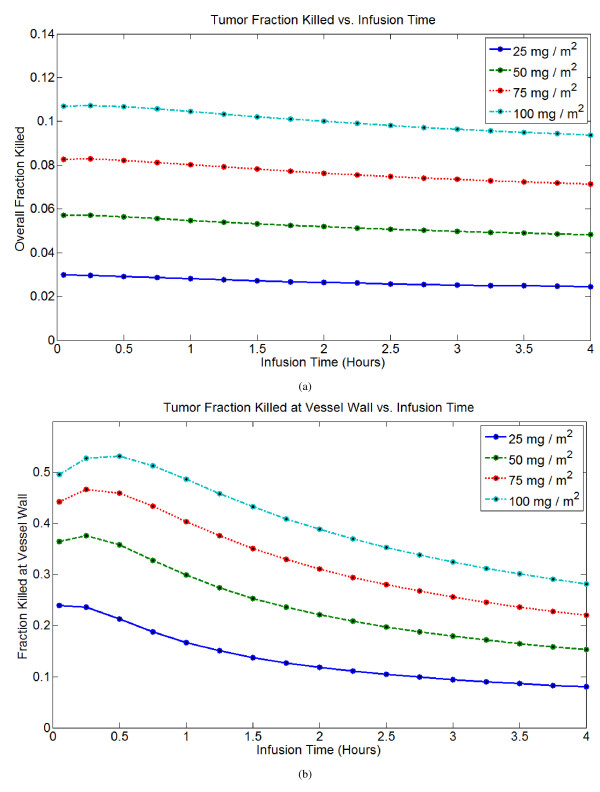
**Metrics of treatment efficacy for a treatment of 75 mg/m^2 ^delivered for infusion times between 0 and 4 hours (0 hours ~3 minute bolus infusion)**. (A) Tumor fraction killed vs. infusion time. (B) Tumor fraction killed at vessel wall vs. infusion time.

I compare the efficacy of doxorubicin treatment by bolus injection versus continuous infusions. Following treatment, the cell fraction killed at every point is predicted from the peak intracellular concentration, and integrating over the tumor cord gives the total fraction of cancer cells killed. I primarily use two metrics to measure efficacy: the total fraction of cancer cells killed and the fraction of cancer cells killed at the vessel wall. As these metrics are based upon peak intracellular concentration, the intracellular AUC at each spatial point in the tumor cord is also tracked. Overall cell mortality and mortality at the cell wall are strongly, but not perfectly, correlated. Given that *in vivo *greater proliferation and better oxygenation will be seen near the vessel wall, predicted cell kill near the vessel wall may be a better predictor of efficacy than overall cell kill, as the model does not account for these complicating factors. In general, the model predicts that short infusion times (less than 1 hour) are best, and the optimal infusion time depends on the dose. For smaller doses, a rapid bolus is optimal, while for larger doses, infusion times up to about 1 hour are as effective or better than bolus injection. For infusions longer than 2 hours, there is a significant reduction in efficacy. The spatial profile of cell kill within a tumor cord for a single dose of 75 mg/m^2^under different infusion times is shown in Figure 4, and Figure 5 gives overall cell mortality and mortality at the vessel wall as a function of infusion time for several different doses.

**Figure 6:**
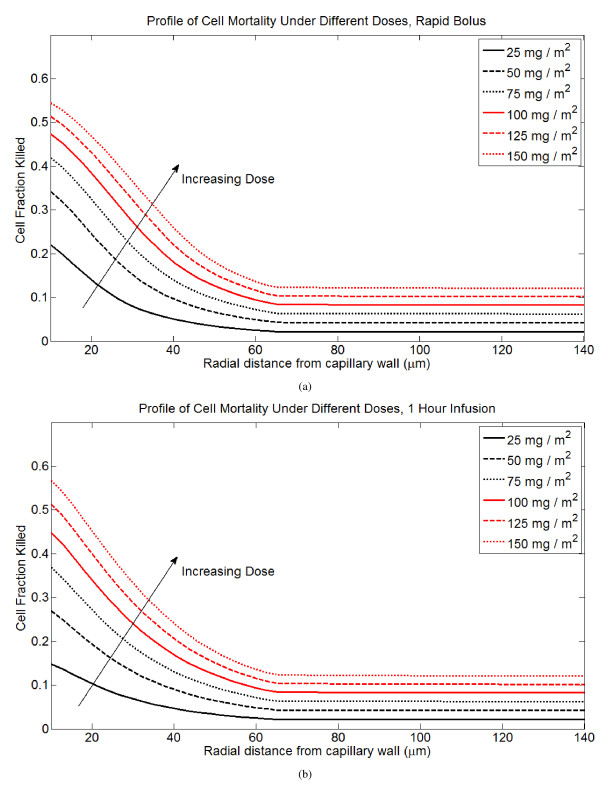
**Profiles of cell mortality under rapid bolus and 1 hour infusion for different doses of DOX, ranging from 25 mg/m^2 ^to 150 mg/m^2^**. (A) Rapid bolus. (B) 1 hour infusion.

I examine the efficacy of low-dose (LD) versus high-dose (HD) chemotherapy delivered in a single infusion to a tumor cord. With increasing dose, cell mortality at the vessel wall increases semi-linearly, and total cell mortality increases linearly. Profiles of cell mortality under different doses are shown in Figure 6.

### Treatment under different pharmacokinetic parameters

The pharmacokinetic parameters describing DOX plasma dynamics are well-described by a 3-compartment model, but the parameters vary significantly between patients. Robert *et al. *[20] measured short-term response to DOX treatment in 12 breast cancer patients and compared pharmacokinetic parameters to response, finding that plasma AUC was a poor predictor of response. Using the reported pharmacokinetic parameters and dose for each patient, I have run a simulated course of therapy in a tumor cord for each patient (all other parameters are the baseline values given in Table 1). The predicted tumor fraction killed and mortality at the vessel wall both correlate well with the reported response following a single treatment, indicating that the model has some utility in predicting responses to DOX therapy.

I have performed a sensitivity analysis of the 6 plasma pharmacokinetic parameters, *A*, *B*, *C*, *α*, *β*, *γ*, by varying each over the parameter range in [20]. The model predicts that *A *and *α*, which determine the kinetics of the initial distribution phase, are the most important parameters in determining both overall cell mortality and mortality at the vessel wall. This is also in accord with the results of Robert *et al.*, who found a strong correlation between *A *and the short-term tumor response, and a moderate correlation between *a *and the short-term tumor response.

The fraction of DOX that is bound to plasma proteins is also important in determining DOX delivery to the tumor cord. As expected, increasing the fraction of plasma drug that is free significantly improves delivery and cell kill. Unexpectedly, however, this is not the case for free extracellular DOX, and increasing the fraction bound actually increases cell kill. Bound extracellular drug apparently acts as a reservoir during the elimination phase and limits clearance out of the tumor to the vasculature.

### Treatment under different microenvironment parameters

To give a picture of how doxorubicin delivery varies in different microenvironments, a sensitivity analysis on all the parameters that describe the tumor cord geometry and transport to the cord has been performed.

#### Plasma to cord transport

The parameters determining convective flux, i.e. hydraulic conductivity, *L*_*p *_and the hydrostatic and osmotic pressure gradients, Δ_P _= *P*_*V*_-*P*_*E *_and ΔΠ = Π_*E*_-Π_*V*_, have only a small effect on the transport of DOX to the tumor cord, at least within what has been determined to be biologically realistic parameter space. Thus, the transport of DOX appears to be dominated by diffusive rather than convective forces, and elevated tumor IFP is only a minor barrier to treatment by doxorubicin.

The diffusive permeability of free DOX, *P*_*F*_, is extremely important in determining transport to the tumor cord, and is likely the single most important parameter. The diffusive flux of bound DOX is nearly negligible, and increasing *P*_*B *_increases transport by an insignificant amount. Thus, diffusive transport of free DOX is the dominant mechanism by which the drug is delivered to the tumor cord.

#### Extracellular diffusion

Altering the diffusion coefficients for free and bound extracellular DOX have different effects on delivery. Increasing diffusion for free DOX (*D*_*F*_) significantly increases drug delivery. Increasing diffusion for bound DOX (*D*_*B*_) actually inhibits delivery slightly and reduces cell kill near the vessel wall. I interpret this to mean that the dominant effect of high diffusion for free DOX is to reduce the drug concentration near the vessel wall during the plasma distribution phase and therefore aid transport into the tumor cord. During the terminal phase the DOX gradient inverts and drug begins clearing back into the vasculature, and the dominant effect of high diffusion for bound DOX is to aid in this clearance.

#### Cell packing

The baseline cell density (*d*_*C*_) and fraction extracellular space (*ϕ*) significantly affect drug delivery. Examining these parameters independently of each other suggests that increases in cell density inhibit transport, yet a lower fraction extracellular space aids transport. However, these variables are related by the relationship



where *V*_*C *_is the volume of a single cancer cell. Examining the effect of these two variables when constrained by this relationship indicates that increasing the fraction extracellular space (and thus reducing cell density) increases the overall cell kill in the tumor cord, but reduces cell kill at the vessel wall. Therefore, it can be concluded that tighter cell packing increases drug sequestration and mortality near the cell wall, but inhibits the transport of drug deeper into the tumor cord. Looser cell packing results in a more uniform profile of mortality with overall mortality greater.

#### Vessel and cord radii

**Figure 7:**
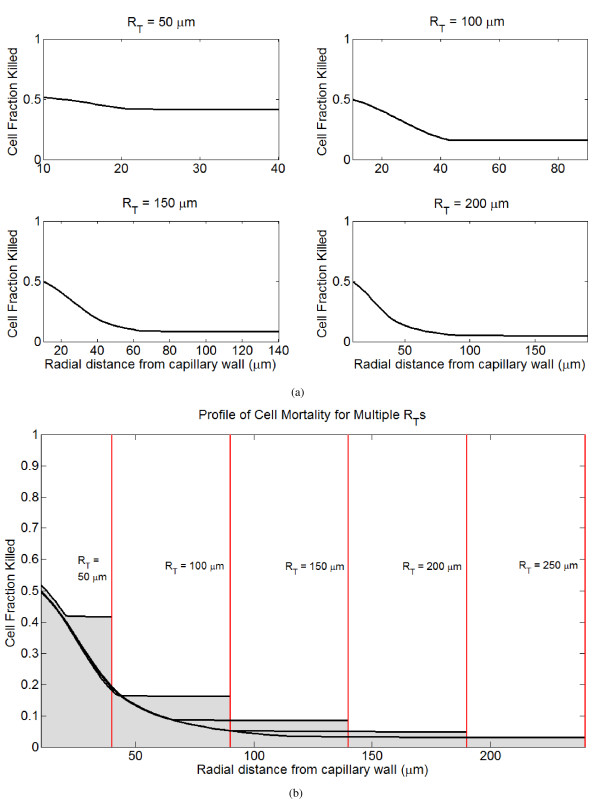
**Profiles of tumor cell mortality for a single infusion of 75 mg/m^2 ^delivered as a rapid bolus in tumor cords with different radii (*R*_*T*_)**. (A) Spatial profiles of cell kill for different tumor cord radii. (B) Spatial profiles of cell kill for different tumor cord radii superimposed in the same plot.

The tumor cord radius, *R*_*T*_, is a function of the intercapillary distance in the tumor, and the reflecting boundary condition at the outer edge of the tumor cord reflects the effect of DOX diffusing from neighboring tissue. The tumor cord radius dramatically affects DOX delivery to the cord system, with smaller cords experiencing much greater cell kill deeper within the cord. Interestingly, the profile near the vessel wall is not greatly affected by *R*_*T*_. Profiles of cell mortality following treatment for different tumor cord radii are shown in Figure 7. Increasing the tumor capillary radius, *R*_*C*_, results in improved drug delivery as measured by all metrics. The capillary radius also affects the optimal infusion time. Larger radii increase the efficacy of continuous infusions, while tumors with small radii respond better to bolus injection. Since the abnormal tumor vessels are typically dilated [48], this result supports the use of continuous infusions in advanced tumors.

### Multiple treatments

**Figure 8:**
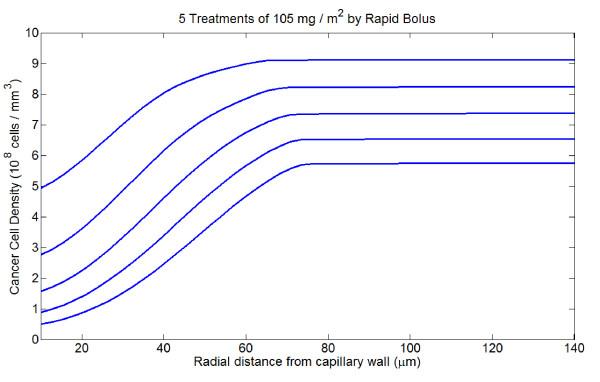
**Spatial profiles of tumor cell density over the course of five treatments of 105 mg/m^2 ^by rapid bolus**.

I simulate the application of several subsequent treatments. Each treatment is run as a separate simulation, and following each treatment cell mortality everywhere in the tumor cord is calculated. From this, a new, spatially explicit profile of cancer cell density is calculated which is used as the initial condition for *C*(*r*) for the next treatment. Also, the fraction of tumor extracellular space, *ϕ*, is recalculated at every point according to the relation



where *V*_*C *_is the volume of a single tumor cell. No cancer regrowth between administration of subsequent treatments is considered, nor is the effect of cell migration into space freed by cell death, but these should be addressed in the future. All treatments are equally efficacious in terms of total cell mortality, although the relative cell mortality at the vessel wall is generally greatest for the initial treatment. The profiles of surviving cells after each treatment for 5 bolus infusions of 105 mg/m^2 ^are shown in Figure 8.

Simulating the delivery of a total dose of 525 mg/m^2 ^as either 10 doses (52.5 mg/m^2^), 7 doses (75 mg/m^2^), or 5 doses (105 mg/m^2^) suggests that greater fractionation gives slightly better results. Furthermore, response to multiple treatments strongly depends upon the tumor cord radius: delivering 525 mg/m^2 ^results in a 50% regression in a tumor cord with a radius of 150 *μ*m, 74% regression for a radius of 100 *μ*m, and nearly 100% regression for a radius of 50 *μ*m. This is comparable to breast tumor regressions of between 10 and 95% (average 74%) following 5 courses of doxorubicin (50 mg/m^2 ^each), vincristine, and methotrexate combination therapy reported by Robert *et al. *[20]. For tumor cords with large radii (likely advanced tumors), mortality beyond 100 *μ*m from the vessel wall is limited even after multiple treatments. However, if cell motility were to be taken into account, it is possible that the surviving cancer cell population could shift towards the vessel wall. This would likely increase the efficacy of subsequent treatments. Because of their increased activity at the vessel wall, short continuous infusions would then, perhaps, be relatively more effective. The space-filling effect of necrotic debris and clearance of this matter into the circulation probably also plays a role. Therefore, these results can only be viewed as preliminary.

### Minimizing peak plasma concentration

Because peak plasma concentration of DOX is correlated with cardiotoxicity and other side effects, there has been interest in reducing cardiotoxicity either by increasing infusion time or dividing single large infusions into multiple smaller infusions. For example, Greene *et al. *[22] proposed that dividing a 15 minute infusion of 75 mg/m^2 ^into 5 infusions of 15 mg/m^2 ^lasting 120 minutes, peak plasma concentration could be reduced 30-fold without reducing plasma AUC. I examine the efficacy of such fractionated regimes compared to single infusions.

**Figure 9:**
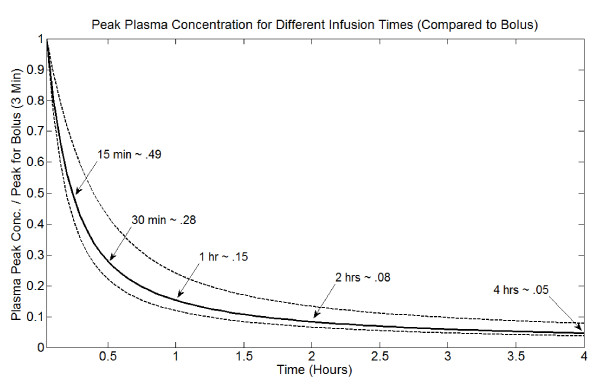
**Peak plasma DOX concentration for different infusion times, relative to rapid bolus (3 minutes)**. This curve depends on pharmacokinetic parameters; the center curve is the average for the 12 parameter sets reported by Robert et al. [20]. The minimum and maximum curves from this data-set are also shown.

Figure 9 shows how the peak plasma concentration changes with infusion time, relative to a rapid bolus of 3 minutes. This depends upon an individual's plasma pharmacokinetic parameters, and the range for the 12 patients reported by Robert *et al. *[20] is displayed along with the average. Peak plasma concentration changes linearly with dose (*D*). In general, dividing a dose given by rapid bolus into several smaller doses (that independently cause mortality) slightly increases the efficacy of the treatment. Thus, the previous result for fractionating a total dose of 525 mg/m^2 ^scales down. However, the efficacy of different infusion times changes with dose size, with rapid bolus better for smaller doses. Therefore, the two strategies for reducing peak plasma concentration are "competing" to some degree. For example, giving a 75 mg/m^2 ^dose as a 1 hour infusion reduces peak plasma concentration nearly 7-fold (see Figure 9); a 2 hour infusion reduces it 12-fold. Dividing a 75 mg/m^2^bolus into five 15 mg/m^2 ^boluses alone reduces peak concentration 5-fold. I have found that for this dose size, 30 minute infusions preserve anti-tumor activity, but overall, peak plasma concentration is reduced 18-fold compared to a single bolus of 75 mg/m^2^. Extending to a 1 hour infusion decreases efficacy somewhat, but peak plasma concentration is reduced over 30-fold. Thus, fractionating doses and using brief infusions (no longer than 1 hour) is likely a better strategy than extended infusion times for a single dose. However, both are effective, and a single extended infusion may be more practical clinically.

## Conclusion

The essence of my model is the conceptual coupling of the earlier doxorubicin uptake pharmacokinetics model of El-Kareh and Secomb [3], the plasma pharmacokinetics model of Robert *et al. *[20], and the well-known principle of solute transport [21]. Applying this model to a tumor cord geometry gives a relatively simple framework that allows realistic modeling of drug delivery in an *in vivo *tumor. This model allows quantification of the behavior of doxorubicin within the spatial environment of the tumor. Cell mortality can be predicted, and all parameters can be estimated directly from empirical data and their importance quantified.

Cardiotoxicity is widely believed to be related to peak plasma concentration [6,22,24], and several clinical trials have demonstrated that, in adults, long-term infusion (24–96 hours) of doxorubicin has reduced cardiotoxicity compared to bolus injection [6,49,50].

This leads to the most important and clinically relevant result of the paper, which is that cardiotoxicity may be reduced while maintaining anti-tumor efficacy through two dose scheduling strategies: (1) Extend infusion time (up to 2 hours) for the standard dose (50–75 mg/m^2^), (2) Fractionate the standard dose into several smaller infusions (15–25 mg/m^2^). The latter strategy has previously been suggested by several groups [22,24]. For smaller doses, infusion times less than 1 hour preserve anti-tumor activity and further reduce peak plasma concentration. Moreover, while peak plasma concentration is reduced dramatically for short infusions, the reduction in peak concentration decreases with increasing infusion time (see Figure 9). Thus, combining the two strategies to deliver several small doses infused for short times may be optimal.

Results also suggest that DOX transport to tumor cords can be characterized by two phases; the first phase primarily determines cell mortality near the tumor cord's vessel wall, and the second establishes a relatively uniform "baseline" level of mortality. To maximize cell kill, it is essential to maximize DOX delivery during the first phase. For small doses of the drug, this is best accomplished by a rapid bolus. For larger doses, continuous infusions are slightly more efficacious. The importance of the first (distribution) phase is supported by sensitivity analysis of the plasma pharmacokinetic parameters, which suggests that *A *and *α *are the most important parameters in determining cell kill within the tumor cord. Robert *et al. *[20] also found these parameters to be the most important in predicting tumor response.

Results indicate that the tumor microenvironment is important in determining drug delivery into solid tumors, and the diffusional permeability of free DOX, the effective diffusion coefficient of free DOX, cell packing density, the tumor capillary radius, and overall tumor cord radius are all significant determinants of DOX delivery to the system. Denser tumors display increased cell kill near the vessel wall, while less dense tumors have more uniform delivery that penetrates further into the tumor cord. Since the effective diffusion coefficient is likely to increase with the fraction extracellular space, less dense tumors may respond better to chemotherapy.

Somewhat surprisingly, the tumor IFP is relatively unimportant, and lowering it increases treatment efficacy by only a small amount. Thus, the model predicts that elevated tumor IFP does not pose a significant barrier to DOX treatment. This makes sense in light of the model's other prediction: transport to the tumor cord is dominated by diffusive transport free DOX.

Interestingly, binding to proteins affects DOX delivery differently in the plasma and extracellular tumor space. While increasing the fraction of bound DOX in the plasma inhibits delivery to the tumor cord, increasing binding to proteins in the extracellular space causes bound DOX to act as a reservoir that increases cellular exposure to DOX.

Increasing the vessel radius greatly increases the transport of DOX into the tumor cord; this is expected, as doing so increases the vascular area of exchange. Increasing the tumor cord radius, and hence the intercapillary distance within the larger tumor, dramatically decreases the efficacy of DOX treatment.

For larger tumor cords (radius ≥ 150 *μ*m), cell kill beyond about 100 *μ*m from the vessel wall is low, even for an infusion of 150 mg/m^2^. Deep within the cord, cell kill increases with additional treatments, but ultimately does not exceed 50% even for a cumulative dose of 525 mg/m^2^. Therefore, limited distribution due to large intercapillary distances may represent a fundamental mechanism by which advanced tumors resist chemotherapy, at least in the case of doxorubicin. This mechanism has been suggested by other authors as well [5,7,51]. However, for small tumor cords (radius ≈ 50 *μ*m) cell kill is much greater throughout the tumor cord, and overall mortality approaches 100% for a cumulative dose of 525 mg/m^2^.

There are several important limitations to the model. While I have found that elevated IFP is not a major barrier to transcapillary DOX transport, elevated tumor IFP can cause fluid to flow out of the tumor and leads to washout of cytotoxic drugs. This has not been taken into account in this model, but has been studied previously with mathematical models by Baxter and Jain [9-11]. There are also several active metabolites of doxorubicin that have not been modeled. They have less cytotoxic activity than DOX [52], and total plasma exposure is less for the metabolites than for DOX itself [22]. Therefore, disregarding the metabolites of DOX is a reasonable first approximation, but they may still play some role in determining optimal infusions. The distribution of oxygen or other nutrients from the tumor capillary and the role of these factors in mediating cell density and regrowth following treatment can and should be incorporated into the model, as in the work by Bertuzzi *et al. *[13].

This model framework has potential for expansion. Different anti-tumor agents could easily be considered by incorporating other existing pharmacokinetic models (see [4,53]), and the efficacy of different combination treatments could be easily evaluated. This tumor cord geometry can also be coupled to complex, multi-organ system pharmacokinetic models, such as the model for doxorubicin developed by Harris and Gross [54]. Finally, the tumor cord microarchitecture could be used in a modeling framework where a whole tumor is viewed as an aggregation of tumor cords. Tumors are heterogeneous, and the parameters describing these tumor cords are expected to vary depending on location. Therefore, delivery to multiple cords described by different parameters could be simulated and the results aggregated to predict overall response to therapy.

## Competing interests

The authors declare that they have no competing interests.
